# Integration of a Copper-Containing Biohybrid (CuHARS) with Cellulose for Subsequent Degradation and Biomedical Control

**DOI:** 10.3390/ijerph15050844

**Published:** 2018-04-25

**Authors:** Anik Karan, Margarita Darder, Urna Kansakar, Zach Norcross, Mark A. DeCoster

**Affiliations:** 1 Cellular Neuroscience Laboratory, Biomedical Engineering, College of Engineering and Science, Louisiana Tech University, Ruston, LA 71270, USA; aka029@latech.edu (A.K.); uka003@latech.edu (U.K.); norcrosszachary@gmail.com (Z.N.); 2 Materials Science Institute of Madrid (ICMM), CSIC, 28049 Madrid, Spain; darder@icmm.csic.es; 3 Cellular Neuroscience Laboratory, Institute for Micromanufacturing, College of Engineering and Science, Louisiana Tech University, Ruston, LA 71270, USA

**Keywords:** biohybrids, cellulose, degradation, nanomaterials, cell culture, green materials, composites, copper

## Abstract

We previously described the novel synthesis of a copper high-aspect ratio structure (CuHARS) biohybrid material using cystine. While extremely stable in water, CuHARS is completely (but slowly) degradable in cellular media. Here, integration of the CuHARS into cellulose matrices was carried out to provide added control for CuHARS degradation. Synthesized CuHARS was concentrated by centrifugation and then dried. The weighed mass was re-suspended in water. CuHARS was stable in water for months without degradation. In contrast, 25 μg/mL of the CuHARS in complete cell culture media was completely degraded (slowly) in 18 days under physiological conditions. Stable integration of CuHARS into cellulose matrices was achieved through assembly by mixing cellulose micro- and nano-fibers and CuHARS in an aqueous (pulp mixture) phase, followed by drying. Additional materials were integrated to make the hybrids magnetically susceptible. The cellulose-CuHARS composite films could be transferred, weighed, and cut into usable pieces; they maintained their form after rehydration in water for at least 7 days and were compatible with cell culture studies using brain tumor (glioma) cells. These studies demonstrate utility of a CuHARS-cellulose biohybrid for applied applications including: (1) a platform for biomedical tracking and (2) integration into a 2D/3D matrix using natural products (cellulose).

## 1. Introduction

Nano- and micro-scale materials are increasingly being introduced into our environment, including the broad areas of biomedical materials and non-biomedical materials [1,2,3,4]. One major concern about these materials is their ability to be degraded over time [5,6]. For biomedical materials, unless the material is to be a long-lasting implant [7] or external body covering such as a bandage, the fate of the materials used should be of concern as far as clearance by the body and/or possible inflammatory response [8,9]. For non-biomedical materials, nano- and micro-scale substances may be “out-of-sight” to many of us, but not actually disappear from the environment, as we have seen in the case of plastic microparticles [10,11].

We previously described the novel synthesis and initial characterization of a self-assembled copper-containing biohybrid with the amino acid dimer cystine that forms high-aspect ratio structures [12,13]. We named this biohybrid material CuHARS, and it has both nano- and micro-scale features. Since CuHARS is self-assembled in a bottom up manner from aqueous solution, we hypothesized that a natural product material compatible with liquid integration might be used to construct stable matrices incorporating CuHARS and other materials for CuHARS delivery. Herein, we show successful integration of CuHARS and other nanomaterials with the natural matrix substance cellulose.

Cellulose is the most abundant polysaccharide on earth. The fibers present as structural components in plants, tunicates, algae, and certain bacteria are formed by aggregated chains of β-1,4-linked d-glucose units. Diverse chemical and mechanical methodologies have been proposed to defibrillate or fragment these fibers leading to microfibrils, elementary fibrils, or nanocrystals [14]. Among these processes, the 2,2,6,6-tetramethylpiperidine-1-oxyl radical (TEMPO)-mediated oxidation of cellulose followed by mechanical disintegration is commonly employed to obtain individualized fibrils [15,16], which can be later assembled with other nano- and micro-materials to develop a wide variety of renewable composite substances. For instance, functional or multifunctional materials for diverse purposes have been reported combining cellulose nanofibers with clay minerals [17], carbon nanotubes [18], or nanoparticles with magnetic [19] or antimicrobial properties [20]. Additionally, cellulose-based materials show great potential for biomedical applications in drug delivery and wound healing, as scaffolds for tissue engineering and in vitro testing of cell models, and in other medical uses [21,22,23].

In the current studies, we have demonstrated the effective degradation of CuHARS under physiological conditions, and compatibility with cells under in vitro conditions. Thus, a non-toxic, biocompatible stable matrix (cellulose) can be utilized to construct and deliver fully degradable novel copper containing biohybrids for biomedical or potentially other applications using fully natural products.

## 2. Materials and Methods

### 2.1. Materials and Reagents

Whatman filters (grade-1) 32 mm with 11 µm pores were obtained from VWR (Radnor, PA, USA, catalogue#: 1001-032). TEMPO (2,2,6,6-tetramethylpiperidine-1-oxyl (98%)), sodium bromide, sodium hypochlorite (reagent grade, available chlorine 10–15%), Fe_2_O_3_ nanoparticles (<50 nm), copper sulfate, cystine, copper oxide nanoparticles (average size = 50 nm), and NaOH were obtained from Sigma-Aldrich (St. Louis, MO, USA).

### 2.2. Synthesis of CuHARS

CuHARS were handled and synthesized as previously described using copper sulfate and cystine [12,13]. Synthesized CuHARS mixtures were transferred to 15 mL or 50 mL centrifuge tubes for concentration. To ensure that any precipitates of copper not associated with CuHARS were destroyed, 3 µL of 0.1 N HCl was added per 10 mL of CuHARS solution. This mixture was then vortexed for 1 min to dissolve any precipitates. The tube was then centrifuged at 3000× *g*, the supernatant decanted, and sterile water added with vortexing to wash the pellet. The washing and centrifugation process was repeated a third time, and then as much of the supernatant was removed as possible without disturbing the pellet. For determination of mass, this wet pellet of CuHARS was then transferred to a hot plate by adding CuHARS material to a glass microscope slide and dehydrating. Dried material can be removed from the glass surface and weighed to calculate the yield and mass of the final product. Using these methods, and as described previously [12,13], synthesis of CuHARS resulted in materials that range from 20 nm and larger in diameter, and hundreds of nm and more in length. 

### 2.3. Generation of Cellulose Films and Cellulose Hybrid Materials

Cellulose fibers were obtained by TEMPO mediated oxidation from Whatman filters following the procedure described by Saito et al. [15]. Briefly, the filters (1 g) were suspended in 100 mL of water and converted into a pulp using a domestic blender, and then 16 mg of TEMPO and 100 mg of sodium bromide were added to the pulp under magnetic stirring at 400 rpm. The TEMPO mediated oxidation was started by adding 9 mL of sodium hypochlorite solution dropwise, and the pH was maintained at 10 by adding 0.5 M NaOH solution. The process was maintained for 4 h. The TEMPO-oxidized cellulose fibers were then centrifuged at 9000× *g* relative centrifugal force (rcf) for 4 min. The resulting fibers were resuspended in water and centrifuged as above as a washing step and this process was repeated twice. Next, the fibers were resuspended in water to a concentration of around 0.4% *w*/*v*, and blended for about 1 h to produce the mechanical defibrillation of the cellulose pulp. Finally, the suspension was centrifuged at 2000 rcf for 4 min in order to remove the thicker fibers. After this process, the final concentration of the suspension was 0.3% *w*/*v*. The cellulose suspension was stored at 4 °C before further experiments were carried out. The carboxyl content was determined by means of conductometric titration with 0.04 M NaOH, using a Pt conductivity cell 50–70 and GLP 31 conductivity meter from Crison, and it was found to be 1.41 mmol carboxylic groups per gram of cellulose.

The biohybrid cellulose/nanoparticles films were prepared by mixing the cellulose fiber dispersion with diverse nanomaterials to construct solid biohybrid films by simple casting and drying. Cellulose/CuHARS materials were prepared by dispersing 0.5 mL of a 1 mg/mL CuHARS solution in 4 mL of 0.3% *w*/*v* cellulose suspension. The mixture was vortexed for homogenization, and 4–5 mL/well was then placed in one or more of the wells of a 12 well suspension plate (Greiner) and dried in the oven at 37 °C for 48 h to produce cellulose/CuHARS biohybrid films. The content of CuHARS nano- and micro-materials in the films is about 4% of the total mass. Similarly, cellulose/CuHARS/Fe_2_O_3_ materials were also prepared by addition of 0.5 mL of previously sonicated Fe_2_O_3_ nanoparticles (1 mg/mL solution) together with the CuHARS into the cellulose dispersion. In this case, the films were loaded with 3.8% CuHARS and 3.8% Fe_2_O_3_ nanoparticles with respect to the total mass. For comparison of CuHARS with copper nanoparticles, cellulose-based materials containing 4% of the total mass of copper oxide nanoparticles (CuONPs) were prepared following the same procedure as described for cellulose/CuHARS composite films.

### 2.4. Measurement of CuHARS Degradation

Passive CuHARS breakdown (in the absence of cells), was evaluated under physiological conditions (37 °C and 5% CO_2_) in 4 different brain/neuronal cell culture media. All media evaluated contained complete growth components including serum. The evaluated media included: (1) CRL-2303 (brain tumor, glioma) cell media, prepared as previously described [24] and as suggested by the vendor for CRL-2303 cells (ATCC, Manassas, VA, USA); (2) Primary brain astrocyte cell culture media, prepared as previously described [24], and included both fetal bovine serum (FBS) and horse serum; (3) Microglial cell media, prepared as suggested by the vendor (ATCC) for the microglial cell line CRL-3265; (4) PC-12 cell media, prepared as suggested by the vendor (ATCC for cell line: CRL-1721). Degradation of CuHARS was measured from digital microscopy images using ImageJ software (version 1.5b, developed by NIH and freely available at: imagej.nih.gov/ij/).

### 2.5. Cell Culture

Cellulose films incorporating CuHARS, CuONPs, and CuHARS + Fe_2_O_3_NPs were combined with CRL-2303 brain tumor (glioma) cells plated at 20,000 cells/well on 24-well cell culture plates as indicated. Cells were allowed to attach to well plates, and were incubated for up to 74 h under physiological conditions at 37 °C and 5% CO_2_, in humidified incubators. 

### 2.6. Digital Microscopy Imaging

CuHARS alone and CuHARS biohybrids incorporated into cellulose matrices and incubated with cells were imaged using a Leica DMI 6000B inverted microscope (Leica Microsystems, Wetzlar, Germany). Since the cellulose matrix biofilms were slightly above the Z-axis focal plane compared to the underlying cell culture area, images shown for matrix cell interactions were composed by carrying out a digital overlay function using Adobe Photoshop (Version 6.0.1, Adobe Systems, Inc., San Jose, CA, USA). A digital image focused at the Z-plane of the cellulose matrix and another digital image focused at the plane of the cells were combined by digital overlay.

## 3. Results

### 3.1. Degradation of CuHARS Materials

**Figure 1:**
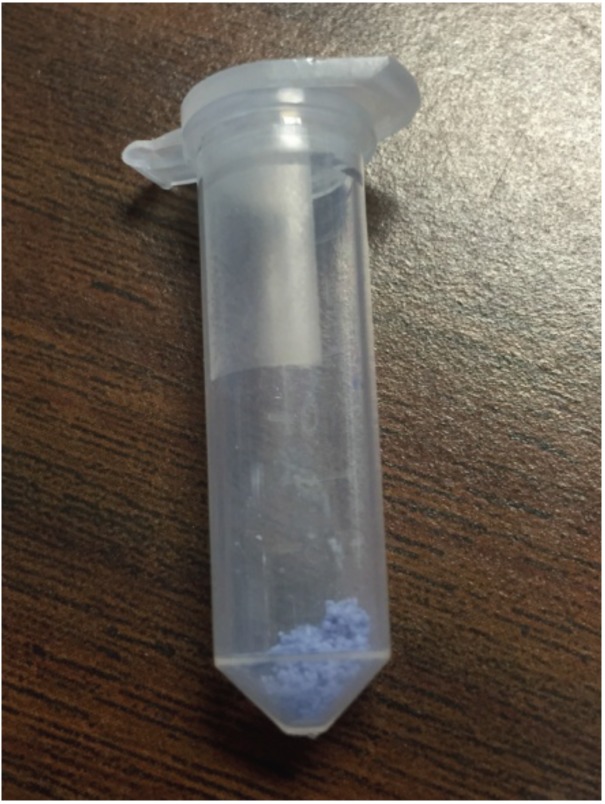
Dried CuHARS product. CuHARS was synthesized and dried as indicated in Section 2. A yield of 5 mg of blue product is shown in the bottom of a 2 mL plastic tube.

Since reporting the discovery and novel synthesis of CuHARS materials [12,13], we have observed over time that post-synthesis, the material is very stable in water, and through a process of centrifugation for concentration and low heat drying (below 100 °C, see Section 2), may be produced in milligram quantities (Figure 1).

**Figure 2:**
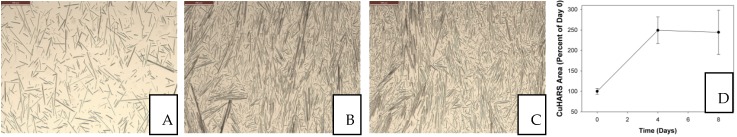
Stability of CuHARS in water. CuHARS at 25 µg/mL in water was added to a 48 well cell culture plate (Greiner) and observed at 0, 4, and 8 days using white light microscopy (panels **A**, **B**, and **C**, respectively). Scale bar in upper left of panels (**A**–**C**) = 100 microns. The area of CuHARS coverage for multiple fields and multiple wells over time was calculated using ImageJ image analysis software (Panel (**D**)). Data shown are the averages for 9 analyzed fields for each day with standard error of the mean shown.

In our experience from synthesizing the CuHARS, we have observed that the material is very stable in the synthesis vessels, with no indication of material breakdown for months when stored under refrigerated conditions. To quantify this observation directly, we measured the change in CuHARS area coverage over a period of 8 days at room temperature, and found no degradation (Figure 2). In fact, CuHARS in this environment was extremely mobile, and from day 0 through day 8, retracted from the test well edges towards the center between days 0–4, and stabilized thereafter (Figure 2C).

**Figure 3:**
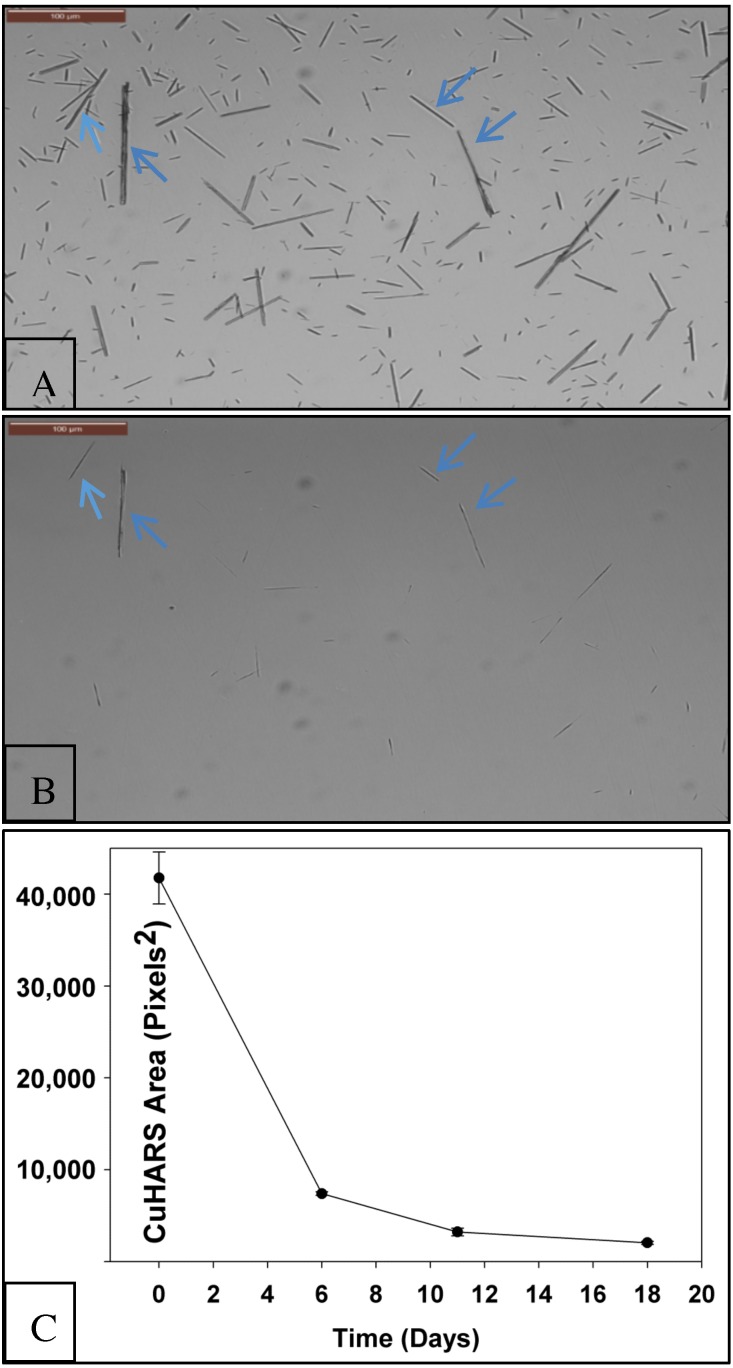
Passive breakdown of CuHARS under physiological conditions. CuHARS at 25 μg/mL was placed in complete microglial cell media (see methods), but with no cells present. Panel (**A**) shows CuHARS area coverages after 1 day in vitro, and in Panel (**B**), near complete breakdown of materials after 18 days. The two panels shown are the same area tracked over 18 days. Only the largest CuHARS remain after this time (arrows). Scale bar in upper left indicates 100 microns. Monochrome images are shown here to enhance the contrast of the highly degraded (thin) CuHARS. Panel (**C**): digital images of CuHARS at 25 μg/mL starting concentration (day 0) were obtained in 4 different neuronal cell culture media (see Section 2), and the area of CuHARS quantified at days 0, 6, 11, and 18, as indicated. Data shown are the averages of fields tracked in 4 different culture media over 18 days, with standard error of the mean shown.

In contrast to this behavior in water, CuHARS slowly, but nearly completely, broke down when placed in cell culture media at 37 °C for a period of 18 days (Figure 3A,B). Compared to the starting area coverage of CuHARS at day 0, the CuHARS area was decreased by over 95% by day 18 (Figure 3C).

### 3.2. Cellulose Matrix Compositions for Holding CuHARS and Other Materials

**Figure 4:**
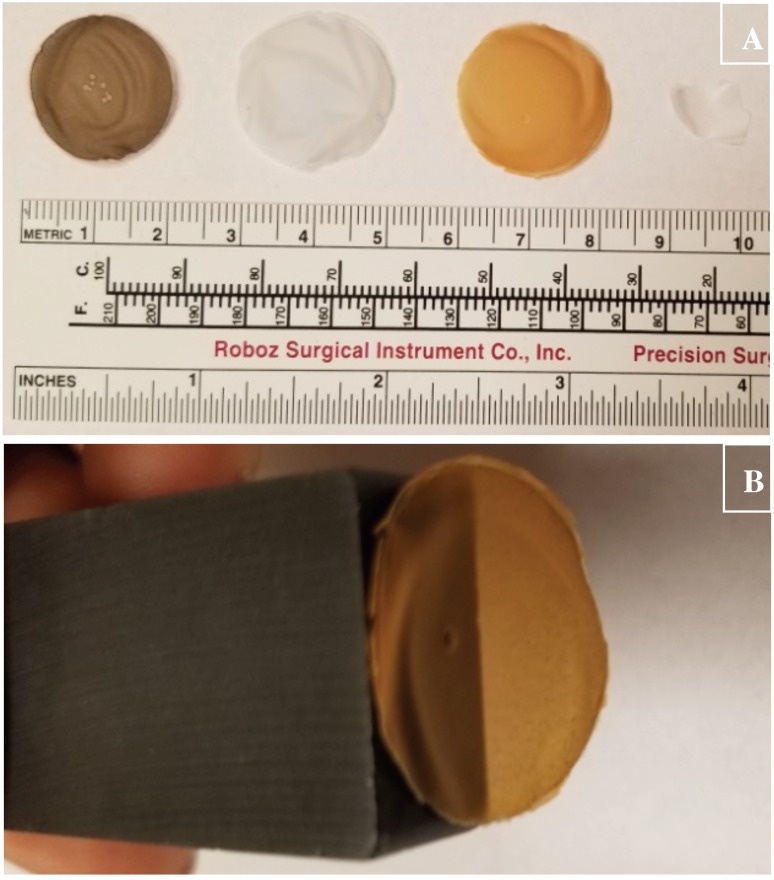
Cellulose matrices for materials integration. Panel (**A**) cellulose matrix at the macroscale, showing from left to right, cellulose biohybrids incorporating CuONPs, CuHARS, Fe_2_O_3_NPs/CuHARS, and cellulose alone, respectively. Metric scale below the matrix discs indicates a diameter of approximately 2 cm for each disc. The 4th sample (rightmost sample) is a fragment of the constructed cellulose matrix alone. Panel (**B**) magnetic susceptibility of materials incorporating Fe_2_O_3_NPs/CuHARS. Cellulose matrix sample incorporating susceptible Fe_2_O_3_NPs is suspended by a permanent bar magnet; the diameter of disc is same as shown in panel (**A**) (=2 cm).

The stable and non-aggregating properties of CuHARS led us to hypothesize that incorporation of these materials into a porous, but non-degrading matrix could be useful to spatially control CuHARS delivery and CuHARS density in 2- and 3-dimensions. We tested this hypothesis by constructing CuHARS-cellulose matrices in a liquid/pulp phase, and then drying them into stable, solid films which could be easily handled (Figure 4A). Furthermore, biohybrids using the cellulose matrix and incorporating both Fe_2_O_3_NPs and CuHARS remained magnetically susceptible to a permanent bar magnet for at least 8 months after fabrication of the material (Figure 4B).

**Figure 5:**
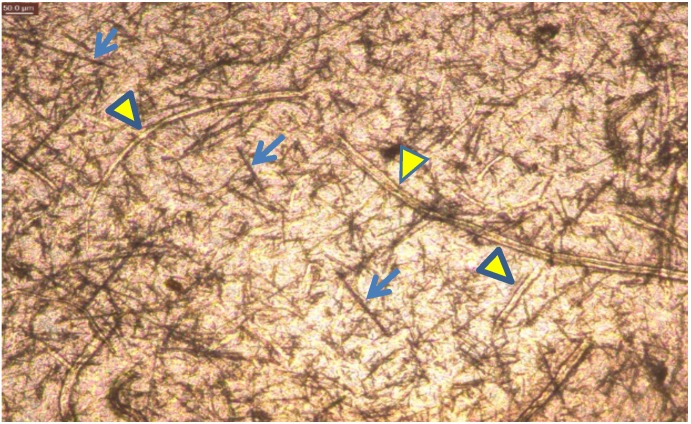
Cellulose matrices for material integration. Cellulose and CuHARS together. Dark, straight structures are CuHARS (blue arrows), and light, curved structures are cellulose fibers (yellow arrowheads). Image obtained using inverted light microscopy, with a 50 micron scale bar indicated (top left of image).

Microscopic digital imaging of the constructed biohybrid cellulose matrices demonstrated curved, more transparent cellulose fibers integrated with straight, dark high-aspect ratio structures which were identified as the CuHARS component (Figure 5). As we previously showed [12], the copper component of CuHARS provides sufficient contrast using white-light microscopy such that the material stands out well against white light backgrounds. This was confirmed in the current work upon integrating CuHARS into cellulose matrices, which were sufficiently thin to pass light, and reveal both cellulose fibers and the straight, dark contrasting CuHARS components (Figure 5). Although we are not using cellulose nanofibers that produce highly transparent films, the type of cellulose used in this work gives rise to films with a sufficient degree of transparency for visualization using inverted white light microscopy. A 0.1% suspension of this cellulose shows a transmittance of around 30% at 600 nm due to light scattering, as it is composed of a mixture of micro- and nano-fibers (data not shown). Nevertheless, its preparation has the advantage of being less time and energy consuming than for nanofibers, and it gives rise to films with enough quality for the purposes demonstrated here.

### 3.3. Application of Constructed Cellulose Materials to Aqueous Solutions

**Figure 6:**
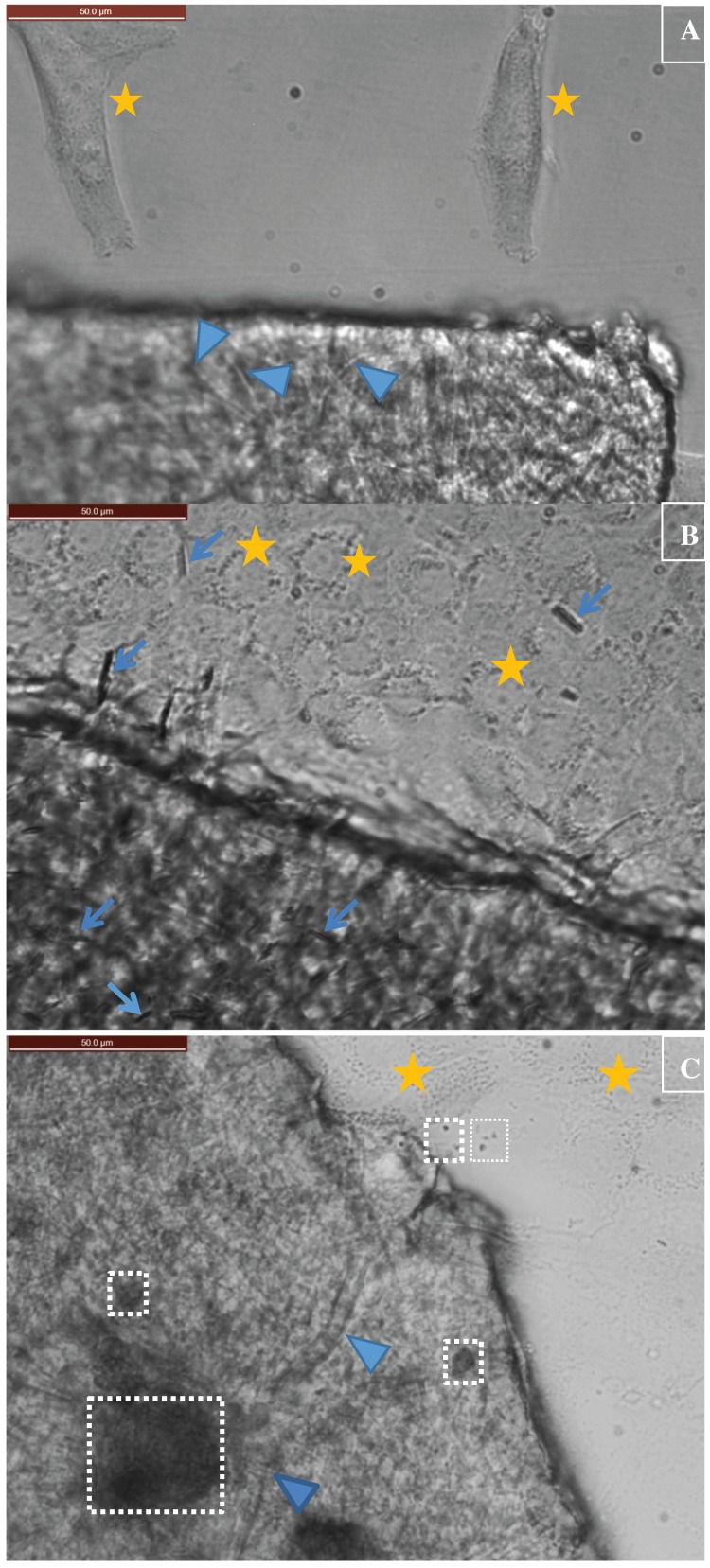

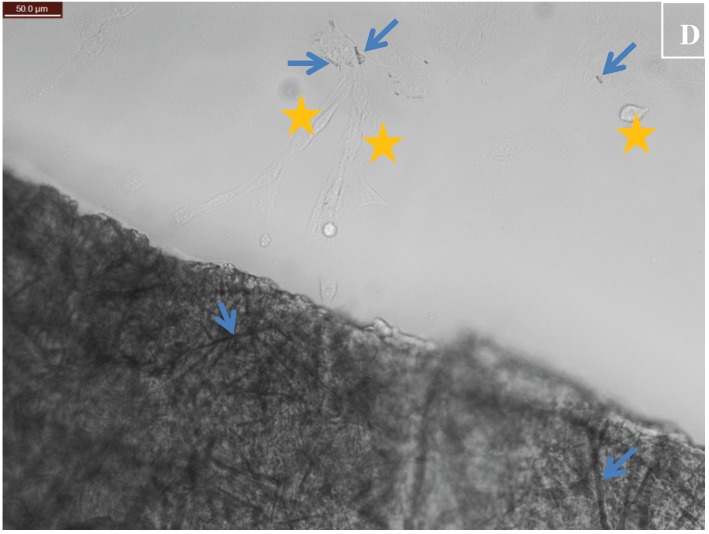
(**A**) Cellulose film testing with cells in defined microenvironments. Cellulose matrix materials alone were tested for compatibility with cell culture conditions. Bottom portion of the image shows the cellulose matrix and top portion shows glioma cells. Scale bar indicates 50 microns (upper left of figure). Blue arrowheads indicate cellulose fibers and stars indicate edges of glioma cells; (**B**) Cellulose films testing with cells in defined microenvironments. Cellulose-CuHARS biohybrids. Bottom portion shows the cellulose-CuHARS matrix and top portion shows glioma cells with some released CuHARS. To evaluate integration into the matrix of smaller size biohybrids, CuHARS were sonicated as previously described [12] before combining with cellulose. Scale bar indicates 50 microns (upper left of figure). Blue arrows indicate CuHARS and stars indicate edges of glioma cells; (**C**) Cellulose-CuONPs biohybrids. Left and lower portion shows the cellulose-CuONPs matrix and right and upper portion shows glioma cells with some released CuONPs aggregates. Scale bar indicates 50 microns (upper left of figure). Blue arrowheads indicate cellulose fibers, stars indicate edges of glioma cells, and white boxes indicate CuONPs aggregates of different sizes; (**D**) Cellulose-CuHARS-Fe_2_O_3_NPs biohybrids. Bottom portion shows the cellulose-CuHARS-Fe_2_O_3_NPs matrix and top portion shows glioma cells with some released CuHARS. Scale bar indicates 50 microns (upper left of figure). For these integrated hybrids, CuHARS were not sonicated before mixing with cellulose. Therefore, some very long CuHARS were successfully integrated into the cellulose matrix, as indicated. Blue arrows indicate CuHARS and stars indicate edges of glioma cells.

The stability of constructed cellulose materials for potential environmental and biomedical applications in aqueous environments was tested by placing pieces of constructed films into water and cell culture media. Films were successfully constructed which provided material properties that were wettable and also were easily immersible in the liquid of interest (non-floating). The stability of constructed films in aqueous environments led us to next test if the CuHARS-cellulose and other constructed films were compatible with in vitro cell culture systems. As shown in Figure 6, fast-growing brain tumor (glioma) cells were used as a model for assessment, and demonstrated that constructed films could be placed within cell culture plates for testing purposes. Since the cellulose films are easily wettable, but still somewhat mobile within the media-containing wells, the cut pieces of film still move around to some extent. Additionally, since the films settle on top of the cells, images were constructed from these in vitro experiments by digitally overlaying the Z-plane of focus for the films with the Z-plane focus for the cells, for the same field of view for a given image (Figure 6A–D).

## 4. Discussion

In the present study, we quantified the passive and slow (but complete) degradation of CuHARS in complete cell culture media and under physiological conditions (but in the absence of cells). This property contrasts with other foundational materials such as CuONPs which we had previously shown to rapidly aggregate and degrade under similar conditions, and which were significantly toxic to cells at 25 µg/mL [13]. In contrast to CuONPs, CuHARS materials are largely non-aggregating in many aqueous conditions and less toxic to cells than CuONPs [13], which allowed for us to quantify degradation over the same microscopic fields over many days by quantifying the area of CuHARS using digital image analysis (Figure 3). Using this same technique, we found that CuHARS in water is highly mobile, actually retracting from the test plate well edges and concentrating in the centers of each well tested from days 0–4, and then remaining stable thereafter (Figure 2). Because we observed that the CuHARS remained laterally highly mobile over the observation period, this effect may be a “reverse-coffee-ring” effect, whereby high-aspect ratio structures suppress the coffee-ring effect observed for small, symmetrical particles [25].

In contrast to the long-term stability of CuHARS in water for months which we observed using our standard storage conditions (data not shown), the nearly complete (but slow) breakdown of CuHARS in complete cell media under physiological conditions (Figure 3) may be partly explained due to metal chelating properties of typical culture medias. Since all the media that we evaluated in this work contained fetal bovine and/or horse sera, likely copper binding proteins to be considered could include ceruloplasmin [26], which is a serum ferroxidase that contains more than 95% of the copper found in plasma, and albumin, which also has copper-binding properties [27]. 

Degradation of CuHARS as demonstrated here in cell culture media was considered a positive feature of the synthesized materials. This result led us to the question of whether the biohybrid materials could be stably incorporated into a non-degradable matrix so that the CuHARS could be presented at defined densities and locations and then released in a controlled manner. Here we tested this idea under aqueous conditions and found that the natural product cellulose was an ideal matrix for CuHARS integration as it is non-biodegradable in vivo, or at best, slowly degradable [23]; others have also used the term “biodurable” when referring to cellulose interaction in vivo [28]. Furthermore, the microfibers of cellulose used here were determined to still maintain a considerable degree of crystallinity confirmed by X-ray diffraction (data not shown), and amorphous (non-crystalline) forms of cellulose have shown preferred degradation in vivo over crystalline forms [23]. Initially, two major beneficial aspects were found for CuHARS integration with cellulose: (1) biohybrid sheets could be constructed that when dried, provided sufficient light penetration for effective imaging of materials by white light microscopy (Figure 5); and (2) cellulose matrices alone (Figure 6A), and CuHARS-cellulose and other integrated materials were sufficiently wettable to interact with cells for initial in vitro testing (Figure 6B–D).

For further proof of principle for comparative purposes with CuHARS, we also integrated magnetically susceptible Fe_2_O_3_NPs into cellulose matrices and also CuONPs into cellulose (Figure 4A). In the case of Fe_2_O_3_NPs, the generated material retained magnetic susceptibility for at least 8 months after fabrication with storage under dry, room temperature conditions (Figure 4B). Since we had previously shown that CuHARS materials are less toxic than CuONPs towards brain tumor (glioma) cells [13], CuHARS integrated into cellulose matrices may provide a delayed action delivery platform to cells, including cancer cells, in contrast to the “burst” or immediate effect that might be anticipated from nanoparticles (including copper nanoparticles). Because of the poor survival outcomes in brain tumor patients, new experimental treatments are still actively being sought, including the use of Gliadel wafers [29], which are used as an alternative materials delivery method for local chemotherapy delivery in the brain. As a potential solution to a more external biomedical question, the antimicrobial effect of silver-impregnated cellulose has already been evaluated [30], and was shown to have low toxicity to human cells, while still killing microbes. Finally, due to the stability and scalability of CuHARS-cellulose matrices shown here, and including incorporation of additional functional materials such as Fe_2_O_3_NPs, environmental and sensor applications may be possible. For both biomedical and non-biomedical needs, cellulose matrices are anticipated to provide an important, environmentally benign foundational material, which is also of a renewable nature [31], generated from biomass, and available from one of the most abundant biopolymers on the planet.

## 5. Conclusions

CuHARS may be integrated into a matrix containing the natural product cellulose, forming a convenient biohybrid. The CuHARS-cellulose biohybrid composite may also be combined with magnetically susceptible materials such as Fe_2_O_3_NPs for further control of the materials, and the same cellulose matrix format can be used to generate stable matrix discs incorporating CuONPs. The generated cellulose matrix discs are sufficiently thin to pass light for white light microscopy imaging and are sufficiently wettable and compatible with cell culture media to permit interactions for in vitro testing purposes. Since CuHARS materials are completely (but slowly) degradable under physiological conditions, incorporation of CuHARS into cellulose matrices may extend degradation lifetimes for applications including biomedical and environmental delivery and sensing.

## Abbreviations

CuHARS	Copper containing High Aspect Ratio Structures
CuONPs	Copper Oxide Nanoparticles
FBS	fetal bovine serum
Fe_2_O_3_NPs	Iron Oxide Nanoparticles
rcf	relative centrifugal force
TEMPO	2,2,6,6-tetramethylpiperidine-1-oxyl radical
